# Cesarean delivery among women who gave birth in Dessie town hospitals, Northeast Ethiopia

**DOI:** 10.1371/journal.pone.0216344

**Published:** 2019-05-06

**Authors:** Awoke Giletew Wondie, Atinkut Alamirrew Zeleke, Hedija Yenus, Gizachew Assefa Tessema

**Affiliations:** 1 Department of Reproductive Health, Debre Tabor University, Debre Tabor, Ethiopia; 2 Department of Health Informatics, Institute of Public Health, University of Gondar, Gondar, Ethiopia; 3 Department of Reproductive Health, Institute of Public Health, University of Gondar, Gondar, Ethiopia; Bielefeld University, GERMANY

## Abstract

**Background:**

One of the key strategies for reducing maternal and perinatal morbidities and mortalities is the provision of skilled intrapartum care. While cesarean section is an important emergency obstetric intervention for saving the lives of mothers and newborns, a study comparing the prevalence of cesarean delivery is not sufficiently available in Ethiopia. This study aimed at assessing the prevalence and associated factors of cesarean delivery among women who gave birth at hospitals in Dessie town, Northeast Ethiopia.

**Methods:**

A facility based cross-sectional study was conducted between July and October 2013. A total of 520 women who gave birth in four hospitals (public = 1, private = 3) were interviewed. Face-to-face interviews using a pre-tested and structured questionnaire were conducted for primary data collection. Additionally, patients’ charts were reviewed to collect mothers’ clinical data. Bivariate and multiple logistic regressions analyses were conducted. Odds ratios and 95% confidence intervals were computed and a P-value of less than 0.05 was taken to declare the level of significance.

**Results:**

A total of 512 mothers were included in the final analysis (response rate = 98.4%), the prevalence of cesarean delivery was found to be 47.6% (95% CI: 44.3, 51.1), While 46 (18.2%) of the procedure conducted in public and 198 (76.1%) were in private hospitals. Partograph monitoring [AOR = 3.84 95%CI: 2.24, 6.59], oxytocin administration [AOR = 4. 80 95%CI: 2.87–8.02], previous cesarean delivery [AOR = 2. 86 95%CI: 1.64–5.01] and place of delivery being a private hospital [AOR = 6. 79 95%CI: 4.18–11.01)] were associated with cesarean delivery.

**Conclusion:**

The prevalence of cesarean delivery was found to be high, and was significantly higher in private hospitals than a public facility. There is a need to conduct cesarean delivery audits to appropriately utilize scarce resources. Further an in-depth exploration of the experiences of women with cesarean delivery is necessary.

## Introduction

Maternal mortality remains the global challenge with 275,288 deaths occurring due to pregnancy and complications in 2015 and it was unacceptably high in low- and middle-income countries particularly in sub-Sahara African countries [1]. Similarly, despite the Ethiopian health sector has shown some progress in terms of reducing under-five mortality, the rate of maternal mortality has shown minimal or insignificant change in the last decades [2]. This could be attributed to the low level of maternal health services utilisation during pregnancy and childbirth. In respect to this, the Ethiopian Demographic and Health Survey (EDHS) reported that more than three-quarters of women are still not attending health facilities for delivery [3].

Even at times when women visited health facilities during delivery, quality health care services that included Emergency Obstetric and Newborn Care (EmONC) for possible life-threatening complications are not properly provided. In order to detect and early referral before these complications develop, health facilities must have a functioning basic emergency obstetric and newborn care (BEmONC) which include the seven basic signal functions such as parenteral (intravenous, intramuscular) antibiotics, anticonvulsants, oxytocics, manual removal of placenta, removal of retained products, assisted vaginal delivery and neonatal resuscitation. Moreover, a health facility with a properly functioning comprehensive EmONC (CEmONC) which mainly include cesarean delivery and blood transfusion services on top of the services under the BEmONC is required to prevent avoidable maternal and neonatal mortality [4].

Cesarean delivery is a surgical intervention designed to prevent or treat life-threatening maternal or fetal complications [4]. The World Health Organization (WHO) estimated that about 5–15% of pregnancies would face life-threatening complications [5–8]. When it is done on a timely basis, cesarean delivery provides a golden opportunity to prevent the adverse obstetric outcomes, including maternal death, stillbirth and nearly neonatal death, obstetric fistula, uterine prolapse and sexual dissatisfaction [9, 10]. However, health facilities equipped with necessary instruments and skilled provider are limited in low-and-middle income countries including Ethiopia [11]. At the same time, it has also been observed that the cesarean rate is growing globally for several medical and non-medical reasons [12]. Besides the medical recommendations, evidence has shown cesarean deliveries have been performed either by the mothers' demand or the professional business based malpractices [13–16]. Unnecessary cesarean deliveries can increase the risk of maternal mortality, maternal morbidity, and higher fetal mortality, higher numbers of babies admitted to intensive care [17–21]. These problems could have resulted from the fact that cesarean incision is associated with risks of severe blood loss, postoperative adhesions, incisional hernias and wound infections [22].

The appropriateness and ethical aspects of on-demand cesarean delivery have been a debatable issue among experts in the field [23]. The rate of cesarean delivery had shown disparities in different parts of the world. A cesarean delivery rate in 137 countries revealed that there was a difference in the rates of cesarean deliveries among countries. In the study, while 54 countries, the rate of cesarean delivery was less than 10% in 54 countries, it was between 10–15% in 14 countries, and higher than 15% in 69 countries [24].

The 2016 EDHS found that 2% of live births in the 5 years before the survey was delivered by cesarean section [3]. Another facility reviews for cesarean delivery also showed that 18% of facility deliveries had been occurred by cesarean section. This data varied between from 15% in the public sectors to 46% in the private for-profit sectors [25–27].

In Ethiopia maternal indications accounted for 66% and fetal indications for 34% of cesarean deliveries; this percentage did not vary by sector. The primary indications for cesarean were cephalopelvic disproportion (CPD; 34%), fetal distress (15%), and breech/multiple gestations/malpresentation (14%), previous scar (11%) and placenta previa/prepartum haemorrhage (7%) [27]. Another study in Southwest Ethiopia reported the leading indications for cesarean section were: cephalopelvic disproportion (32.3%); previous cesarean section (24.2%); fetal distress (18.1%); malpresentation and malposition (8.8%); and antepartum hemorrhage (4.2%) [28].

This study aimed at assessing the prevalence and associated factors of cesarean delivery among mothers who gave birth at private and public hospitals in Dessie town.

## Methods

### Study setup and population

An institutional-based cross-sectional study was conducted between July and October 2013 in one public hospital—Dessie Referral Hospital and three private hospitals—Ethio General Hospital, Bati General, and Selam General Hospital in Dessie town, Northeast Ethiopia. These hospitals were selected since they were the only hospitals providing cesarean delivery services for seven million catchment population at the time of the study.

### Sample size and sampling procedure

During sample size determination, we have taken both the first and the secondary objectives. After calculating the sample sizes, we have taken the one who gave us the maximum sample size. Firstly, for the first objective, we used single population proportion formula using the assumptions; the proportion (P) = 18% of cesarean delivery from the national review of cesarean section in Ethiopia, 95% confidence level of Za/2 = 1.96, 5% of absolute precision, and a non-response rate of 10%. Moreover, sample sizes were calculated using the factors associated with cesarean delivery, such as malpresentation (14%), and placenta previa (16%) from previous studies [27]. Finally, a total sample size of 520 was considered for the study (Table 1).

**Table 1:** Summary of sample size calculation for all objectives of study.

Objective	Factor /Proportion	Assumptions	Sample size
Objective 1	Proportion	P = 18%, Zα/2 = 1.96, d = 0.05, NP = 10%	305
Objective 2	Placenta previa	OR = 2, P = 16%, Ratio 1:1, power = 80%, CI = 95%, NP = 10%	473
	Malpresentation	OR = 2, P = 14%, Ratio 1:1, power = 80, CI = 95, NP = 10%	520

P = Proportion, OR = Odds Ratio, NP = Non-response

Women who delivered in these four hospitals during the study period were included. In order to invite eligible study participants, stratified sampling technique was employed. In doing so, all hospitals that provided cesarean delivery services stratified as a private (n = 1) and public (n = 3) hospitals. The sample size was then allocated to each hospital proportion considering their average client size in the past six months. Finally, a systematic random sampling technique was employed to recruit participants. In this process, we calculated the k^th^ interval until we got the estimated number of participants.

### Data collection, analysis, and quality control

Pretested and structured questionnaires using face-to-face interviews were used for data collection. The questionnaire was first prepared in English and then translated into Amharic (the local language), and back into English to ensure conceptual consistency (See S1 File). In addition, medical records of women were reviewed by data extraction checklist to collect data on some clinical variables like an indication for cesarean delivery, use of oxytocin, use of partograph, gestational age and instrumental delivery. After providing two days of training on the data collection questionnaire and interviewing techniques, data were collected by four diploma midwives supervised by one public health officer. As women who underwent cesarean delivery were unconscious immediately, we had to invite them for interviewing between 48 and 72 hours of the procedure.

Data were entered using EPI Info version 3.5.3 statistical software and then exported to SPSS windows version 20 for further analysis. Descriptive and summary statistics were used to describe the study population in relation to the relevant variables. Bivariate and multiple logistic regression analyses were fitted to see the effect of each independent variable on the dependent variable. After fitting the bivariate logistic regression model, those variables with a p-value of less than 0.2 were then included in the multiple logistic regression model with the forward stepwise approach. Odds ratios and 95% CIs were computed to see the presence and strength of the association. A p-value of less than 0.05 was used to declare statistical significance.

As a data quality assurance measure, a pre-test was conducted on about 5% of the sample prior to the actual data collection at the University of Gondar Hospital. The appropriateness and clarity of questions were checked and necessary modifications were made in terms of clarifying questions and vague terms. Each item in the questionnaire and checklist was checked by the supervisor and the principal investigator on a daily basis to review the accuracy and completeness of variables. At the completion of data collection, all entered records were inspected for missing or incorrect values and data cleaning and coding cleaned was conducted before analysis by the supervisor and the principal investigator.

### Ethics approval and consent to participate

Ethical clearance was secured from the Institutional Review Board (IRB) of the Institute of Public Health, College of Medicine and Health Sciences at the University of Gondar. Permission to conduct the study was also obtained from South Wollo Zonal Health Office and Medical Directorate Offices of each Hospital. Each study participant was informed about the purpose and objective of the study. Written informed consent was obtained prior to data collection. Privacy and confidentiality were adhered to as the data collection was anonymous.

## Results

### Socio-demographic characteristics of the study participants

Table 2 shows the sociodemographic characteristics of the participants. Of the 520 study samples, 512 women were included in the analysis (response rate = 98.4%). The remaining 8 questionnaires were discarded because of incompleteness. The mean age of the participants was 27.1 years (SD = 3.75) and more than half (52.1%) of them were between the ages of 20–24 years. Two hundred and ninety-one (55%) were from urban areas. Most (94.3%) of the respondents were married. Half (50.9%) were Orthodox Christian in religion. Regarding their educational and occupational status, more than three quarters (81.6%) had attained at least a primary education and 228 (44.7%) were housewives. Moreover, 305(60%) of mothers had a monthly income of less than 2,000 Ethiopian (ETB).

**Table 2:** Socio-demographic characteristics of women who delivered at public and private hospitals in Dessie town, Northeast Ethiopia, 2013 (n = 512).

Variables	Public Hospital	Private Hospitals
	Frequency (%)	Frequency (%)
**Age (years)**		
15–19	7 (2.8)	5 (1.9)
20–24	56 (22.2)	50 (19.2)
25–29	121 (48.0)	146 (56.1)
30–34	56 (22.2)	49 (18.8)
≥ 35	12 (4.8)	10 (3.8)
**Residence**		
Urban	136 (54.0)	145 (55.8)
Rural	116 (46.0)	115 (44.2)
**Religion**		
Orthodox	118 (46.8)	143 (55.0)
Muslim	120 (47.6)	109 (41.9)
Others*	14 (14)	8 (3.1)
**Marital status**		
Married	236 (93.7)	247 (95.0)
Single	3 (1.2)	2 (0.8)
Divorced	13 (5.2)	9 (3.5)
Widowed	0 (0.0)	2 (0.8)
**Educational status**		
Not educated	46 (18.3)	48 (18.5)
Primary	70 (27.8)	80 (30.8)
Secondary	82 (32.5)	73 (28.1)
College or above	53 (21.3)	59 (22.7)
**Monthly income (ETB)**		
< = 1200	90 (35.7)	44 (16.9)
1201–2000	90 (35.7)	81 (31.2)
2001–3000	49 (19.4)	73 (28.1)
> = 3000	23 (9.1)	62 (23.8)
**Occupation**		
Government employee	41 (16.3)	37 (14.2)
Housewife	113 (44.8)	116 (44.6)
Merchant	57 (22.6)	57 (21.9)
Student	14 (5.6)	5 (1.9)
Others**	27 (10.7)	45 (17.3)

* Catholic, protestant

** farmer, daily laborer

### Obstetric characteristics of respondents

Table 3 shows women obstetric and other reproductive health-related characteristics. Two-hundred twelve (41.4%) of the participants gave birth to their first birth. One hundred and forty (27.3%) of them had the previous history of cesarean delivery. Nearly one-third (30%) of them had a history of abortion (both spontaneous and induced). The majority (85.5%) had received antenatal care services among whom more than one-third (37.2%) had four or more visits. Out of all study participants, an instrument (forceps and vacuum) was applied among 330 (64%) mothers to assist their labor. This number includes instrument-assisted laboring mothers who gave birth either vaginally or cesarean delivery because a failed instrumental application has a tendency of leading to cesarean delivery. Moreover, partograph monitoring of labor was done for more than two-thirds (71.3%) of laboring women.

**Table 3:** Obstetric and other reproductive health-related characteristics of respondents in Dessie town hospitals, 2013(n = 512).

Characteristics	Public	Private
	Frequency (%)	Frequency (%)
**Parity**		
First	94 (37.3)	118 (45.4)
Second	83(32.9)	80 (30.7)
Third and above	75 (29.8)	62 (23.8)
History of abortion*		
Yes	85 (33.7)	56(21.5)
No	167 (66.3)	204 (79.5)
**Number of previous abortion (N = 141)**		
One	52 (61.2)	28 (50)
Two	22 (25.8)	22 (39.3)
Three or more	11 (12.9)	6 (10.7)
Previous CS**		
Yes	27 (10.7)	113 (43.4)
No	225 (89.3)	147 (46.6)
ANC visit***		
Yes	200 (79.3)	238 (79.3)
No	52 (21.7)	22 (21.6)
**Number of ANC visit (N = 438)**		
Zero	52 (20.6)	22 (8.4)
One	16 (6.3)	9 (3.4)
Two	50 (19.8)	49 (18.8)
Three	70 (27.7)	81 (31.1)
Four and more	64 (25.4)	99 (38.07)
**Gestational age at delivery**		
< = 36 weeks	52 (20.6)	38 (14.6)
37–39	141 (56.0)	133 (51.2)
40–42	35 (13.9)	77 (29.6)
43 and more	24 (9.5)	12 (4.6)
**Mode of delivery**		
Vaginal	206 (81.7)	62 (23.8)
CS	46 (18.2)	198 (76.2)
**A decision for CS (N = 244)**		
Physician	29 (63.6)	167 (84.5)
Mothers	17 (37.3)	31 (17.7)
**Type of CS (N = 244)**		
Elective	15 (32.6)	86 (43.4)
Emergency	31 (67.3)	112 (56.6)
**Instrumentation applied**		
Yes	159 (63.1)	171 (65.8)
No	93 (36.9)	89 (34.2)
**Apply oxytocin for the current delivery**		
Yes	129 (51.2)	59 (22.7)
No	123 (48.8)	201 (77.3)
**Partograph monitor**		
Yes	218 (86.5)	147 (56.5)
No	34 (13.3)	113 (43.5)

* History of abortion (spontaneous or induced)

** CS Cesarean section

*** ANC Antenatal care for the current pregnancy

### Prevalence of cesarean delivery

In Table 3, the overall prevalence of cesarean delivery was found to be 244 (47.7%) [95% CI: 44.3, 51.1)], where 46(18.2%), and 198(76.2%) of cesarean deliveries had been conducted in public and private hospitals respectively. The decision for the cesarean delivery was made by physicians for 80% of all cesarean delivery. More than half (58.6%) of the cesarean delivery was done as an emergency cesarean delivery.

**Fig 1:**
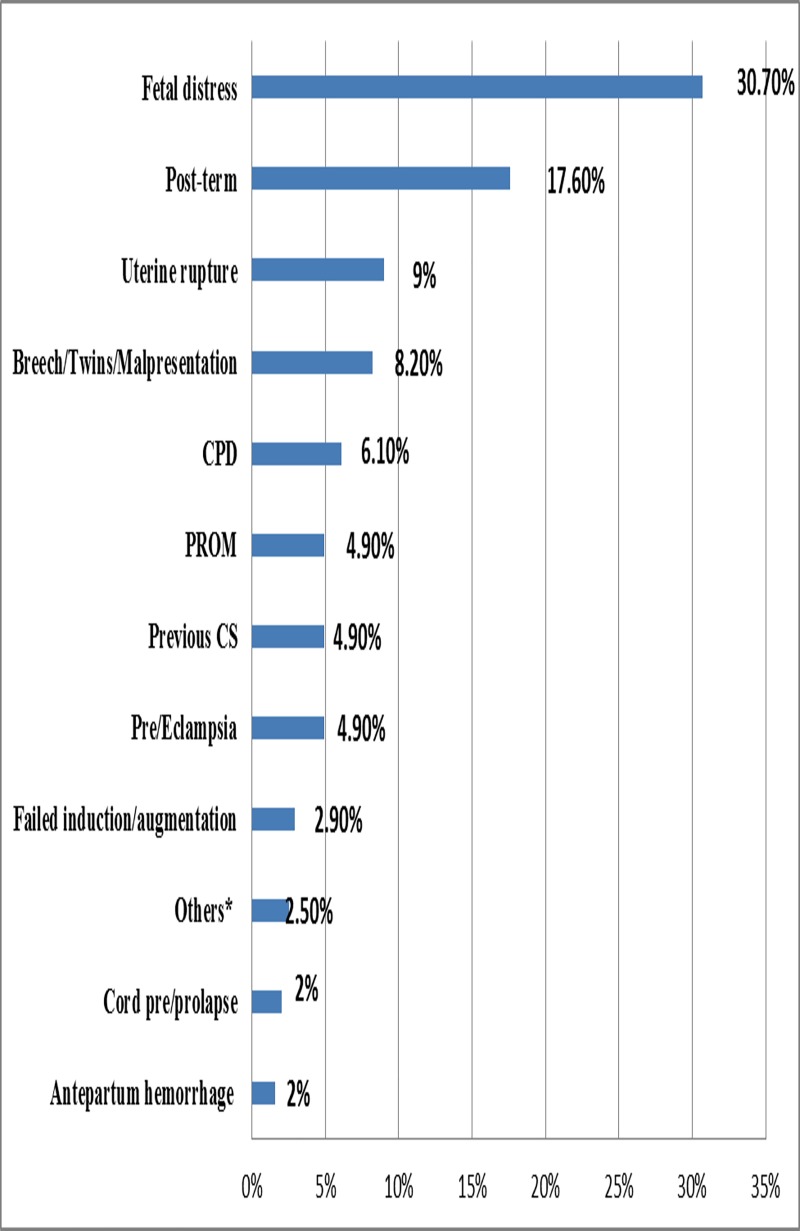
Observed clinical indications for cesarean delivery in both public and private hospitals in Dessie town, Northeast Ethiopia, 2013.

The most common clinical indication for cesarean delivery was fetal distress (30.7%) and post-term pregnancy (17.6%) in both categories of hospitals (Fig 1).

### Factors associated with cesarean delivery

Table 4 shows factors associated with cesarean delivery in hospitals in Dessie town. The bivariate logistic regression analysis identified the type of facility (public vs private), previous cesarean delivery, gestational age, antenatal visit, use of partograph, and oxytocin during labor as a significant variable. However, in multiple logistic regression analysis, type of facility, previous cesarean delivery, partograph utilization, and oxytocin use during labor was significantly and independently associated with cesarean delivery.

**Table 4:** Crude and adjusted odds ratios (OR) and 95% confidence intervals (CI) of factors associated with cesarean section both at hospitals in Dessie town, Northeast Ethiopia, 2013(n = 512).

Variables		Type of delivery		OR (95% CI)			
		Vaginal	Cesarean	COR(95%CI)	P value	AOR(95%CI)	P-value
**Previous cesarean section**							
Absent		229	143	1	0.00	1	0.034
Present		39	101	4.14(2.71, 6.34)		2.86(1.64, 5.01)	
**Oxytocin administered (before delivery)**							
Yes	142		46	1	0.00	1	0.002
No	126		198	4.85(3.25, 7.24)		4.80(2.87, 8.02)	
**Partograph utilization**							
No	49		108	3.54(2.38, 5.29)	0.00	3.84(2.24, 6.59)	0.013
Yes	219		136	1		1	
**Place of delivery**							
Private Hospital	62		198	14.30(9.31, 21.95)	0.002	6.79(4.18, 11.01)	0.020
Public Hospital	206		46	1		1	

Those women having previous cesarean delivery had about three times [AOR = 2.86 (95%CI: 1.64, 5.01)] higher odds of current cesarean delivery as compared to women who had not. In both public and private hospitals the trial of vaginal birth after cesarean section was provided as per the national protocol. Those women who had not been provided oxytocin for induction had about five times [AOR = 4.80 (95%CI: 2.87, 8.02)] more odds of cesarean delivery as compared to women who had received oxytocin during labor. Those women whose labor was not monitored using partograph had about four times [AOR = 3.84 (95%CI: 2.24, 6.59)] more odds of cesarean delivery as compared to those who were not. Regarding the type of institution for delivery, women who attended in private hospitals had about seven times [AOR = 6.79 (95%CI: 4.18, 11.01)] more odds of cesarean delivery as compared to those who attended in a public hospital. None of the socio-demographic characteristics were associated with the outcome variable.

## Discussion

This study was aimed to assess the prevalence and associated factors of cesarean delivery among women who gave birth at hospitals in Dessie town, Northeast Ethiopia. The prevalence of cesarean delivery was found to be 47.6% (95% CI: 44.3, 51.1). The prevalence was higher in private hospitals 76.1% (95% CI: 71.0, 81.5) than the public hospital 18.2% (95%CI: 13.8, 23.0). This finding was higher than the studies conducted in Addis Ababa (5–10%) [25, 26], and a national review of CS in Ethiopia (18%,) which varied between 46% in the private for-profit sector and 15% in the public sector [3, 27]. The difference observed between this study and studies conducted in Addis Ababa could be due to the time difference in the later study conducted two decades ago. This may reflect the relative overtime improvement of access to cesarean delivery in those mothers having labor complications is likely to attend facility delivery in recent years than ever before. Additionally, it could be partly explained by a difference in study setting as studies conducted in Addis Ababa included public hospitals. However, the present study included private hospitals where the likelihood of maternal preferences is acknowledged. Studies conducted in Brazil and Iran suggested that women’s preferences in delivering through CS are higher in private hospitals [29, 30]. The other possible justification would be that Dessie Hospital (the only referral hospital in the catchment population of seven million) receiving referred complicated cases from surrounding health facilities.

In this study, the most common clinical indications for the cesarean section were fetal distress (30.7%) whereas a national review of cesarean section delivery reported cephalo-pelvic disproportion as the main indication for cesarean delivery [27]. All hospitals in this study monitor fetal status by intermittent auscultation using Pinard fetal stethoscope which is a hollow, horn-shaped tube made of wood or metal. With hands-on listening, the care provider listens to the fetal heart rate for short periods of time (at least 60 seconds) at regular intervals (at least every 15–30 minutes during the first stage of labor and at least 5–15 minutes during the second stage of labor). However, most nurses and doctors are not familiar with using a fetal stethoscope and many have little or no training in hands-on listening. This study reported fetal distress as a leading cause of cesarean delivery which could be due to overdiagnosis of fetal distress, in the absence of electronic fetal monitoring system, care providers may not be well trained and hands-on listening may exaggerate the fetal heartbeat since the measurement is subjective and requires the user to be very well trained and acquired a skill in interpreting the results.

Those mothers having a history of previous cesarean delivery had higher odds of having cesarean delivery as compared to those mothers who had not. This finding is supported by studies carried out in Brazil [31, 32]. It could be due to the indication and type of incisions in previous cesarean delivery. The national protocol and the American College of Obstetricians and Gynaecologist (ACOG) suggested that a woman with a history of low-segment cesarean delivery, whose indication is not recurring and who has no contraindication for vaginal birth (e.g. placenta previa, placental abruption with live fetus, abnormal presentation), should be offered a trial of vaginal birth after cesarean delivery (VBAC) in both public and private hospitals. However, most obstetricians are reluctant to give a trial of vaginal birth after cesarean delivery because of the associated significantly high morbidity, mortality, and difficulty in assessing the scar integrity before attempting a trial of labor [33, 34].

Those mothers whose labor were not induced or augmented had more odds of cesarean delivery as compared to those mothers whose labor was induced. This is in line with a study in Brazil and USA [31, 35]. This could be due to the percentage of failed induction (2.5%) that leads to cesarean delivery was observed less frequently. Moreover, this could happen due to the majority of respondents 422(82.4%) in this study reach term gestation (37–42 weeks) and above, spontaneous onset of labor is expected, and for whom oxytocin induction would not be required to initiate labor. On the other hand, for women on trial vaginal birth after cesarean section (VBAC), induction or augmentation of labor is contraindicated because it is associated with an increased risk of scar rupture.

Those mothers whose labor was not monitored using partograph had higher odds of cesarean delivery. In the present study, 55.7% of women had been monitored using the partograph sheet which was higher than a national review of CS in Ethiopia (12%) [27]. This could be due to the status of a laboring mother during admission. A woman could be referred from health institution where no cesarean section service or delayed health service seeking could lead to complications during arrival, for which emergency cesarean delivery would be indicated without monitoring labor.

Regarding the type of institution for delivery, those mothers who delivered at private hospitals had higher odds of cesarean delivery as compared to those who delivered at a public hospital. This finding is in line with a study conducted in Thailand and Brazil [36, 37]. This might be explained by mothers who can afford for private hospital service could prefer elective cesarean delivery due to fear of labor pain. Another possible explanation might be health care providers' poor adherence or practice as per the national protocol in private hospitals.

This study has some limitations. Due to the cross-sectional nature of the study, we did not identify the temporal relationship between cesarean delivery and independent variables. Recall bias might also exist as women may forget previous obstetric related problems. This study is limited as it did not look at the maternal and perinatal outcomes following deliveries.

## Conclusion

The prevalence of cesarean delivery was found to be high. The prevalence is significantly higher in private hospitals. There is a need to conduct cesarean delivery audits to appropriately utilize the scarce resource. Studies comparing the cesarean deliveries between public and private hospitals are required. The present study would also serve as a benchmark for future studies focusing on understanding the experiences of women who undergo cesarean deliveries.

## Supporting information

S1 File Questionnaire and checklist. (PDF)

S2 File Dataset. (SAV)
