# Supplementary material for: Cesarean delivery among women who gave birth in Dessie town hospitals, Northeast Ethiopia
Source: PLoS One. 2019 May 6;14(5):e0216344. doi: 10.1371/journal.pone.0216344 (PMC6502338; doi:10.1371/journal.pone.0216344)
Supplement: S1 File — (PDF) [file pone.0216344.s001.pdf]

# Information sheet

**Title of the research project:** Prevalence and Factors Associated with Cesarean Section in Public and Private Hospitals in Dessie Town, Northeast Ethiopia, 2013.

**Name of the principal investigator**: Awoke Giletew (BSc)

**Name of the organization**: University of Gondar, College of Medicine and Health Science, Institute of Public Health.

**Purpose of research project:** the aim of the study is to assess the prevalence and factors associated with cesarean section in the context of public and private setting in Dessie town.

**Procedure:** Pretested questionnaire was prepared and a woman's having cesarean section or vaginal delivery in selected hospitals will be involved for the study. Since you fulfill the criteria, the team has selected you to be one of the study participants. If you are willing to participate, you are kindly requested to give your genuine response to the data collector during interview.

**Confidentiality:** The information which is collected in the research will be strongly kept confidentially. Which will not have your name on it but the code number will assign to it. And you have the right to refuse from participation. But your honest participation will have contribution to generate valid information that will be used for intervention designs.

**Benefits:** There is no direct benefit to you in participating in this study but it helps us in assessing the prevalence and factors associated with CS among public and private hospitals and to identify gaps.

**Incentive/payment for participating:** you will not be proved any incentives or payment to take part in this project.

**Risk and/or Discomfort:** by participating in this research project you may feel that it has some risks or discomfort but there is no a major risk or discomfort. The interview will only take 15 – 20 minutes.

**Right to refusal or Withdraw:** you have a full right to refuse from participating in this research. You can choose not to answer any or all questions and this will not affect you and your family from getting any type of services. You have also the full right to withdraw from this study at any time you wish, without losing any of your right.

**Persons to contact:** This research project was reviewed and approved by the institutional review board of institute of public health, University of Gondar. If you want to know more information, you can contact the individual and you may ask at any time you want.


1. Awoke Giletew (BSc)
   Cell phone: +2519 18 72 75 86
   E – Mail: awokegiletew@yahoo.com
2. Mr. Atnkut Alamirew(BSc, MPH)
   Cell phone: +2519 11 31 35 78
3. Ms. Hedija Yenus (BSc, MPH)
   Cell phone: +2519 18 81 33 86

## Consent form

**Study questionnaire on prevalence and associated factors of cesarean section among public and private hospitals in Dessie town, Northeast Ethiopia**

*Good morning/good afternoon*

*My name is -----------, I work for ---------------*

*You may have been already informed that a survey on prevalence and factors associated with caesarean section among public and private hospitals will be done this month in Dessie town.*

*This study has been approved by the institutional review board of University of Gondar Institute of public health. Only anonymous data will be analysed. The information which is collected in the research will be strongly kept confidentially. Which will not have your name on it but the code number will be assigned to it.*

*I request you to take part in this study. Your participation is voluntary. If you are willing to participate sign the agreement form. It is possible to stop the interview at any time during the interview process. But your honest participation will have contribution to generate valid information that will be used for intervention designs.You have been randomly selected to participate in this study. The interview is about 15 – 20 minutes long.*

***Signature______*** ***Date____________***

*I will start with the first question.....*

## English version questionnaire

**This questionnaire has 6 parts** which are: Institution characteristics, Socio-demographic characteristics, past obstetric condition, present obstetric condition, and cesarean delivery related information (For women's undergoing C/S). Please strictly follow instruction for each part and try your best to respond to every question accordingly!

**Section 0: Questioner identification**

001-Date_____/_____/_____

002 Name of institution/ Hospital ________________________________________

003- Institution type (Public vs. Private) __________________________________

004-Interviewer Name________________________ Sign_________ date________

005- Result of the interview (please circle it):

1. Completed 2. Incomplete

006- Checked by:

Supervisor Name________________________ Sign_________ date________

**Section1.** ***Socio-demographic and socio-economic Characteristics***

| No | Questions | Alternatives | Remark |
|---|---|---|---|
| 101 | How old are you?<br>In complete years | ______________ | |
| 102 | Where is your residence? | Urban …………….......1<br>Rural ..………………......2 | |
| 103 | What is your religion? | Christian……………......1<br>Muslim ………………......2<br>Protestant………..........3<br>Catholic……….............4<br>other( specify) _________5 | |
| 104 | Your marital status? | Single……………………1<br>Married…………………..2<br>Divorced…………………3<br>Widowed ……………….....4 | |
| 105 | Educational status? | not educated………………1<br>primary………….............2<br>secondary………………….3<br>college and above...............4 | |
| 106 | What is your current occupational status? | Government employee….1<br>Merchant………………….2<br>House wife………………..3<br>Student……………………4<br>Others(specify)………....5 | |
| 107 | What is your family monthly income? | Birr ---------------------------- | |

**Section2. Past obstetric condition**

| No | Questions | Alternative | Remark |
|---|---|---|---|
| 201 | How many times have you had live birth previously? | ------------------ in number | |
| 202 | Did you have previous CS? | Yes----------------------- 1<br>No-------------------------2 | If no, escape to No.304 |
| 203 | If yes, how many times? | ------------------ in number | |
| 204 | Did you have abortion history? | Yes----------------------- 1<br>No-------------------------2 | If no, escape to No.301 |
| 205 | If yes, how many times? | In number ------------- | |

**Section3. Present obstetric condition**

| No | Questions | Alternative | Remark |
|---|---|---|---|
| 301 | Did you have antenatal care follow up in your current pregnancy? | yes----------------------- 1<br>No-------------------------2 | If no, escape to No.303 |
| 302 | If yes, how many times? | In number ------------- | |
| 303 | What was the type of delivery? | C/S-------------------------1<br>Vaginal delivery----------2 | Continue for C/S delivery only. |

**Section4. Cesarean delivery related information (For women's undergoing C/S)**

| No | Questions | Alternative | Remark |
|---|---|---|---|
| 401 | What was the type of CS? | Elective CS --------------------1<br>Emergency CS----------------2 | |
| 402 | Who decide first for need of C/S | Woman-------------------1<br>Physician ----------------2<br>I don't know--------------3 | |

**Thank you**

## Data extraction checklist

### Present obstetric condition

| No | Questions | Alternative | Remark |
|---|---|---|---|
| 501 | What was gestational age during labor and delivery? | ----------------- In completed weeks | Review medical record |
| 502 | Did instrumental device like forceps or vacuums were applied during labor? | Yes --------------------1<br>No----------------------2 | Review medical record |
| 503 | Was there any oxytocin drug administration before delivery? | Yes --------------------1<br>No----------------------2 | Review medical record |
| 504 | Did the labor was monitored using partograph | Yes --------------------1<br>No----------------------2 | Review medical record |
| 505 | What was the primary diagnosis for cesarean delivery | CPD-------------------------------1<br>APH ------------------------------2<br>Twins/malpresentation -------3<br>Fetal distress--------------------4<br>Pre/Eclampsia -----------------5<br>PROM----------------------------6<br>Cord pre/prolapse-------------7<br>Previous CS-------------------8<br>Post term pregnancy--------9<br>Failed induction--------------10<br>RVI-----------------------------11<br>Others -----------------------12 | Review medical record |

## Amharic version information sheet: የመረጃና የፈቃደኝነት ማረጋገጫ ቅጽ

**የመረጃ ገጽ**

**የምርምር ፕሮጀክቱ ርእስ** ፡ በአማራ ክልል ደቡብ ወሎ ዞን ደሴ ከተማ በሚገኙ የመንግስት እና የግል ሆስፒታሎች የሚሰራዉን በሆድ ቀዶ ጥገና የሚወልዱ ሴቶች ላይ ስርጭቱን እና ተያያይዥነት ያላቸው ነገሮችን መዳሰስ ነዉ፡፡

**የዋናው ተመራማሪው ስም** ፡ አወቀ ግለጠው (ጤና መኮንን)

የድርጅቱ ስም ፡ በጎንደር ዩንቨርስቲ ህክምናና ጤና ሳይንስ ኮሌጅ የህብረተሰብ ጤና ትምህርት ቤት

**የጥናቱ ዋና አላማ፡** በአማራ ክልል ደቡብ ወሎ ዞን ደሴ ከተማ በሚገኙ የመንግስት እና የግል ሆስፒታሎች የሚሰራዉን በሆድ ቀዶ ጥገና የሚወልዱ ሴቶች ላይ ስርጥቱን እና ተያያይዥነት ያላቸው ነገሮችን መዳሰስ ሲሆን በሚገኘውም የጥናት ውጤትም ችግሩን ለመቅረፍ የሚያስችሉትን ስልቶችን ማመላከት ይሆናል ፡፡

**አተገባበር**

በሆድ ቀዶ ጥገና የሚወልዱ ሴቶች ላይ ስርጭቱን እና ተያያይዥነት ያላቸው ነገሮች ለማጥናት እርሶዎ በፕሮጀክቱ ተሳታፊ እንዲሆኑ ተጋብዘዋል፡፡በፕሮጀክቱ ለመሳተፍ ፈቃደኛ ከሆኑ ውሉ ሊገባዎትና ሊፈርሙ ይገባል፡፡ ከዚያም በመረጃ ሰብሳቢዎች ጥቂት ደቂቃዎች ለሚወስዱ ጥያቄዎች መልስ እንዲሰጡ በአክብሮት ይጠየቃሉ ፡፡ ለእያንዳንዱ ጥያቄ ለሚሰጡት ምላሽ ማንም በማያገኘው መለያ ቁጥር ሚስጥራዊነቱ የተጠበቀ ይሆናል፡፡

**ምቹ ያልሆኑ ሁኔታዎች፡** በዚህ ጥናት ውስጥ በመሳተፍዎ በተለያየ አልያም ባልታወቀ ምክንያት ምቹ ያልሆኑ ሁኔታዎች ሊያጋጥሙ ይችላሉ፣ይሁን እንጅ ጥናቱ ከሚኖረው ጥቅም አንጻር ሊወዳደር አይችልም

**ጥቅም፡** በዚህ ጥናት በቀጥታ ተጠቃሚ ላይሆኑ ይችላሉ፣ይሁን እንጅ ችግሩን በመቅረፍ ሂደት ዉስጥ ቀጥተኛ ተሳታፊ ነዎት።

**ጉርሻ፡** በዚህ ጥናት ውስጥ በመሳተፍዎ የሚያገኙት ጉርሻ አይኖርም።

**ሚስጥር መጠበቅ፡** የሚሰበሰቡት መረጃዎች ሚስጥራቸዉ የተጠበቀ መሆኑን አረጋግጣለሁ። ለዚህም ሲባል በስምዎ ሳይሆን በሚስጥር ቁጥር ይወከላሉ። በመጠይቁ ሂደት ዉስጥ ለማቆም ከፈለጉ በማንኛዉም ሰዓት በቀላሉ ሊያቆሙ ይችላሉ። ይሁን እንጅ የእርስዎ ሀቀኛ ተሳታፊ መሆን የጥናቱን ቅርጽ በትክክለኛ ወይም ዋጋ ባለው መረጃ የተመሰረተ ለማድረግ ከፍተኛ አስተጽኦ አለው።

**አድራሻ፡** ስለ ጥናቱ መጠየቅ የሚፈልጉት ነገር ካለ የሚከተሉትን አድራሻዎችን መጠቀም ይችላሉ

1. አቶ አትንኩት አላምረዉ፡ ስልክ +251911313578
2. ወ/ሮ ኅድጃ የኑስ ስልክ +251918813386
3. አቶ አወቀ ግለ ጠዉ ስልክ+251918727586

## Amharic version consent: የፈቃደኝነት ማረጋገጫ ቅጽ

**የምርምር ፕሮጀክቱ ርእስ ፡ በአማራ ክልል ደቡብ ወሎ ዞን ደሴ ከተማ በሚገኙ የመንግስት እና የግል ሆስፒታሎች የሚሰራዉን በሆድ ቀዶ ጥገና የሚወልዱ ሴቶች ላይ ስርጭቱን እና ተያያይዥነት ያላቸው ነገሮችን መዳሰስ ነዉ፡፡**

ስሜ________________________________ይባላል።

የምሰራው በ_______________________________ነው።

በዚህ ወር ዉስጥ ደሴ ከተማ በሚገኙ የመንግስት እና የግል ሆስፒታሎች የሚሰራዉን በሆድ ቀዶ ጥገና የሚወልዱ ሴቶች ላይ ስርጭቱን እና ተያያይዥነት ያላቸው ነገሮችን ለመለየት ስለሚደረገው ጥናት ሰምተው እንደሚሆን እገምታለሁ።

ይህንን ጥናት ያረጋገጠው የጎንደር ዩኒቨርስቲ የጥናት ስነምግባር ኮሚቴ ሲሆን፤ የተለኩ መረጃዎች ብቻ ይተነተናሉ። የሚሰበሰቡት መረጃዎች ሚስጥራቸዉ የተጠበቀ መሆኑን አረጋግጣለሁ። ለዚህም ሲባል በስምዎ ሳይሆን በሚስጥር ቁጥር ይወከላሉ።

የጥናቴ ተሳታፊ እንዲሆኑ እየጠየቅኩ፤ ተሳትፎዎ በፈቃደኝነት ላይ የተመሰረተ መሆኑን ለማሳወቅ እወዳለሁ። ፈቃደኛ ከሆኑ በስምምነት ፊርማው ቦታ ላይ ፊርማዎን እንዲያስቀምጡ እጠይቃለሁ። ሆኖም ግን በመጠይቁ ሂደት ዉስጥ ለማቆም ከፈለጉ በማንኛዉም ሰዓት ሊያቆሙ ይችላሉ። ይሁን እንጅ የእርስዎ ሀቀኛ ተሳታፊ መሆን የጥናቱን ቅርጽ በትክክለኛ ወይም ዋጋ ባለው መረጃ የተመሰረተ ለማድረግ ከፍተኛ አስተጽኦ አለው። እርሰዎ ለጥናቱ ያለ ጥቆማ ወይም በነብስ ወከፍ የተመረጡ ናሙና ነዎት። ቃለ መጠይቁ 15-20 ደቂቃ ይወስዳል።

ፊርማ____________________ ቀን_______________

Amharic version questionnaire፡ **የአማረኛ መጠይቅ**

**ክፍል-0 የመጠይቁ መለያ**

001-ቀን_____/_____/_____

002 የሆስፒታሉ ስም______________________________________

003- የሆስፒታሉ እይነት(የመንግስት ወይስ የግል) ___________________________

004- የጠያቂዉ ስም_________________________ ፊርማ__________ቀን_________

005- የመጠይቁ ዉጤት

1. የተሟላ 2. ያልተሟላ

006- ያረጋገጠዉ: ሰዉ ስም_________________________ ፊርማ__________ቀን_________

**ክፍል-1 የእናቶች ማህበራዊና ኢኮኖሚያዊ መረጃዎች**

| ተ.ቁ | ጥያቄዎች | አማራጭ መልሶች | ምርመራ |
|---|---|---|---|
| 101 | ዕድሜ ሽ ስንት ነዉ? | __________________ዓመት | |
| 102 | አድራሻ? | ከተማ----------------------------1<br>ገጠር---------------------------- 2 | |
| 103 | የምን ሀይማኖት ተከታይ ነሽ? | ኦርቶዶክስ ------------------------------- 1<br>እስላም --------------------- ----------- 2<br>ፕሮቴስታን ----------------- ---------- -3<br>ካቶሊክ------------------------------------4<br>ሌላ (ይገለጽ) ----------------------------5 | |
| 104 | የጋብቻ ሁኔታ? | ያላገባች ------------------------------- --1<br>ያገባች -----------------------------------2 | |

| | | | |
|---|---|---|---|
| | | የፈታች-------------------------------------3<br><br>የሞተባት---------------------------------- -4 | |
| 105 | የትምህርት ደረጃሽ? | ማንበብና መጻፍየማትችል ----------------1<br>አንደኛ ደረጃ ያጠናቀቀች ---------------2<br>ሁለተኛ ደረጃ ያጠናቀቀች ---------------3<br>ኮሌጅና ከዚያ በላይ -------------------- -4 | |
| 106 | የስራ ሁኔታ? | የመንግስት ሰራተኛ ---------------------- 1<br>ነጋዴ-------------------------------------- 2<br>የቤት እመቤት --------------------------- 3<br>ተማሪ------------------------------------- 4<br>ሌላ (ይገለጽ) ---------------------------5 | |
| 107 | የቤተሰብ ወርሃዊ ገቢ ለምን ያህል ነዉ? | በብር------------------------------------ | |

## ክፍል-2 ከዚህ በፊት የነበረዉንየእርግዝና እና የፅንስ ሁኔታ የሚያመላከት መረጃ

| ተ.ቁ | ጥያቄ | አማራጮች | ምርመራ |
|---|---|---|---|
| 201 | ከዚህ በፊት ለምን ያህል ጊዜ በህይወት<br><br>የተወለዱ ልጅዎችንወልደዋል? | በቁጥር -------------------- | |
| 202 | ከዚህ በፊት በሆድ ቀዶ ጥገና ወልደዋል? | አዎ-------------------------------------1<br>የለም----------------------------------2 | የለም ከሆነወደ ጥያቄ ቁጥር 204 ይሸጋገሩ |
| 203 | ለጥያቄ 202 መልሱ አዎ ከሆነ ለምን ያህል ጊዜ? | በቁጥር --------------------------- | |

| | | | |
|---|---|---|---|
| 204 | ከዚህ በፊት በነረዉ እርግዝና ውርጃ አጋጥምዎት ያዉቃልን ? | አዎ-------------------------------------1<br>የለም-----------------------------------2 | የለም ከሆነወደ ጥያቄ ቁጥር 301 ይሽጋገሩ |
| 205 | ለጥያቄ 204 መልሱ አዎ ከሆነ ለምን ያህል ጊዜ? | በቁጥር ---------------------------- | |

## ክፍል-3 የአሁኑን እርግዝና እና የዕንስ ሁኔታ የሚያመላክት መረጃ

| ተ.ቁ | ጥያቄ | አማራጮች | ምርመራ |
|---|---|---|---|
| 301 | በአሁኑ የእርግዝና ጊዜ የቅድመ ወሊድ ክትትል አድርግዋል? | አዎ---------------------- -------------1<br>የለም--------------------------------- 2 | የለም ከሆነ ወደ ጥያቄ ቁጥር 303 ይሽጋገሩ |
| 302 | አዎ ከሆነ ለምን ያህል ጊዜ? | በቁጥር ---------------------------- | |
| 303 | የወለዱበት መንገድ | በሆድ ቀዶ ጥገና ------------------------ 1<br>በማህፅን---------------------------------2 | በሆድ ቀዶ ጥገና ለወለዱ ብቻ ይጥሉ |

## ክፍል-4 የወላዱዋ ምርጫ ና ዉሳኔ (በሆድ ቀዶ ጥገና ለወለዱ ብቻ)

| ተ.ቁ | ጥያቄ | አማራጮች | ምርመራ |
|---|---|---|---|
| 401 | በምንአይነት ሆድ ቀዶ ጥገና ነዉ የወለድሽዉ? | በቀጠሮ------------------1<br>ድንገተኛ-------------------2 | |
| 402 | በሆድ ቀዶ ጥገና ለመዉለድ መ ጀመርያ<br>ማን ወስነ? | ወላዱዋ---------------------1<br>ሀኪሙ-------------------------2 | |

# አመሰግናለሁ

# የበሽተኛዉን ካርድ በማየት የሚሞላ መረጃ

| ተ.ቁ | ጥያቄ | አማራጮች | ምርመራ |
|---|---|---|---|
| 501 | በምጥ ወቅት የዕንሱ ዕድሜ ምን ያህል ነዉ? | በሳምንት ይገለጽ ----------- | የበሽተኛዉን ካርድ በማየት የሚሞላ |
| 502 | ለምጥ የሚረዳ መዳኒት ጥቅም ላይ ዉሎአል? | አዎ--------------------- ------------1<br>የለም-------------------------------- 2 | የበሽተኛዉን ካርድ በማየት የሚሞላ |
| 503 | በምጥ ወቅትክትትል ተደርግዋል? | አዎ--------------------- ------------1<br>የለም-------------------------------- 2 | የበሽተኛዉን ካርድ በማየት የሚሞላ |
| 504 | በፍጥነት ለመዉለድ የሚረዳ መሳርያ ጥቅም ላይ ዉሎአል? | አዎ--------------------- ------------1<br>የለም-------------------------------- 2 | የበሽተኛዉን ካርድ በማየት የሚሞላ |
| 505 | በምጥ እናወሊድ ወቅት የተከስቱ የህክምና ምክንያት | የዕንስ መቀርቀር--------------------1<br>የእርግዝና ጊዜ ደም መፍሰስ--------2<br>ትክክል ያልሆነ ዐመጣጥ-------------3<br>የዕንስ መታፈን--------------------4<br>የደም ግፊት ------------------------5<br>የሽርጥ ዉሃ መፍሰስ--------------------6 | የበሽተኛዉን ካርድ በማየት የሚሞላ |

| | | | |
|---|---|---|---|
| | | የእትብት ቀድሞ መምጣት----------------7<br><br>ሌላ -----------------------------------8<br><br>ከዚህ በፊት በሆድ ቀዶ ጥገና---------------------9<br><br>ለምጥ የሚረዳ መዳኒት ጥቅም አልባሲ.ይሆን-----------------------10<br><br>የማህፅንመሰንጠቅ--------------------11 | |
